# Determinants of loss to follow-up in patients on antiretroviral treatment, South Africa, 2004–2012: a cohort study

**DOI:** 10.1186/s12913-015-0912-2

**Published:** 2015-07-04

**Authors:** Mazvita Naome Mberi, Lazarus Rugare Kuonza, Nomathemba Michelle Dube, Cornelius Nattey, Samuel Manda, Robert Summers

**Affiliations:** South African Field Epidemiology Training Programme, National Institute for Communicable Diseases of the National Health Laboratory Services, 1 Modderfontein Road, Monument Park 0105, Post net suite 179, P/bag X27923 Sandringham, South Africa; School of Health Systems and Public Health, Faculty of Health Sciences, University of Pretoria, Pretoria, Dr Savage Road 0084 Pinshof 349, Pretoria, South Africa; Medunsa National Pharmacovigilance Centre, Medunsa Campus, University of Limpopo, Pretoria, South Africa; Wits Reproductive Health and HIV Institute, 22 Esselen Street, Hillbrow, South Africa; National Institute for Occupational Health, Smit Street, Braamfontein, South Africa; Global Partnership Initiated Academia for the control of health threats, Bernard Nocht Institute for Tropical Medicine, Bernad-Nocht Street 74, 20259 Hamburg, Germany; Biostatistics Research unit, South African Medical Research Council, 1 Soutpansberg Road, Pretoria, South Africa; Division of Epidemiology and biostatistics, School of Public Health, University of the Witwatersrand, 27 St Andrews Road Parktown, Johannesburg, South Africa; Department of Pharmacy, Faculty of Health Sciences, MEDUNSA Campus, University of Limpopo, Pretoria, South Africa

**Keywords:** Loss to follow-up, Surveillance cohort, Medunsa National Pharmacovigilance Centre, Antiretroviral therapy

## Abstract

**Background:**

The number of Human Immunodeficiency Virus (HIV) infected people eligible for initiation on antiretroviral Therapy (ART) is increasing. ART programmatic success requires that patients who are taking ART remain on treatment and are followed up regularly. This study investigated factors associated with being lost to follow-up, in a cohort of patients enrolled in a pharmacovigilance study in South Africa.

**Methods:**

This was a retrospective observational cohort study performed at one of the Medunsa National Pharmacovigilance Centre’s (MNPC) ART sentinel surveillance sites. Loss to Follow-up (LTFU) was defined as “a patient who had been followed up at the sentinel site, who had not had contact with the health facility for 180 days or more since their last recorded expected date of return or if there were 180 days or more between the expected date of return and the next clinic visit”.

**Results:**

Out of 595 patients, 65.5 % (*n* = 390) were female and 23.4 % (*n* = 139) were LTFU. The median time on ART before LTFU was 21.5 months (interquartile range: 12.9 – 34.7 months). The incidence rate of LTFU was 103 per 1000 person-years in the first year on ART and increased to 405 per 1000 person-years in the eighth year of taking ART. Factors associated with becoming LTFU included not having a committed partner (Adjusted Hazard Ratio (aHR): 2.9, 95 % Confidence Interval (CI):1.19-6.97, *p* = 0.019), being self-employed (aHR: 13.9, 95 % CI:2.81 - 69.06, *p* = 0.001), baseline CD4 count > 200 cells/ml (aHR: 3.8, 95 % CI: 1.85-7.85, *p* < 0.001), detectable last known Viral Load (VL) (aHR: 3.6, 95 % CI:1.98 - 6.52, *p* < 0.001) and a last known World Health Organisation clinical stage three or four (aHR: 2.0, 95 % CI:1.22-3.27, p = 0.006). Patients that previously had an ART adverse event had a lower risk (aHR: 0.6, 95 % CI: 0.38 - 0.99, *p* = 0.044) of becoming LTFU than those that had not.

**Conclusion:**

The incidence rate of LTFU increases with additional years on ART. Intensified measures to improve patient retention on ART must be prioritised with increasing patient time on ART and in patients that are at increased risk of becoming lost to follow-up.

## Background

Human immunodeficiency Virus (HIV) is a public health challenge worldwide. In 2013, the United Nations program on HIV/AIDS (UNAIDS) reported that there were an estimated 35.3 million people living with HIV globally [1]. The most affected region is Sub Saharan Africa, which accounted for close to 70 % of all new HIV infections in 2013 [2]. South Africa has the highest number of people living with HIV (6.3 million) [3], and also has the largest HIV treatment programme, with 1.8 million persons reported to have been initiated on antiretroviral treatment (ART) since the commencement of the treatment programme [4]. Though there has been an increase in the scale up of access to ART in South Africa, there is also an increasing number of people that are becoming eligible for ART as new ART eligibility criteria are being introduced [4] and newly acquired HIV infections continue to be reported in the population [1].

ART, which continues to be the mainstay of HIV management, acts to prevent HIV multiplication and reduces the viral load in the blood, resulting in improved immune function of an HIV-infected person and a decrease in the risk of transmitting the virus [2, 5]. The accelerated scale up of access to ART has led to a decline in HIV related morbidity, mortality and new HIV infections globally [1, 6]. As sustainability is becoming a priority in the response to the AIDS epidemic, high loss to follow-up (LTFU) rates have the potential to reverse gains that have been achieved thus far.

There is evidence that interrupting ART leads to inferior clinical outcomes and higher risks of opportunistic complications and death in patients [7]. However, studies in sub-Saharan Africa have shown that less than two-thirds of patients initiated on ART are on treatment 2 years after ART initiation [8], and a systemic review showed a 70 % 24 month retention rate and a 65 % 36 month patient retention rate [9]. Furthermore, it has been shown that there is an increasing cumulative incidence of LTFU with each year of taking ART in South Africa [10]. The South African National Strategic Plan on HIV, Tuberculosis and Sexually Transmitted Infections (2012–2016) has a goal not only to initiate at least 80 % of eligible patients on ART, but for at least 70 % of patients that are initiated on ART to be alive and on treatment 5 years after initiation [11].

Though investigations have been done around factors that possibly minimise the rates of LTFU [8, 12–16], few studies have been done to determine factors associated with LTFU in South Africa [17–21]. Coupled with the challenge of limited research around LTFU, there are also several definitions of LTFU which vary across different programs and studies. This means research findings around LTFU may not, necessarily, remain the same if a different definition of LTFU is applied. A study on the impact of LTFU definitions recommended that the definition of LTFU should be based on the study outcome of interest, available encounter data and the cohort visit schedule [22].

The objectives of this study were to determine the incidence of, and factors associated with, LTFU in a cohort of patients enrolled in a Pharmacovigilance (PV) sentinel site in Gauteng province, South Africa.

## Methods

### Study setting

Tshepang clinic is an ART clinic located at Dr. George Mukhari Hospital, one of the largest academic hospitals in Gauteng province. The clinic is one of three functional pilot sites of the Medunsa National Pharmacovigilance Centre’s (MNPC) ART surveillance cohort study. The MNPC was opened in September 2004 as a National Department of Health (NDoH) initiative at the same time when ART became available in the South African public health sector. The Centre operates a structured ART surveillance program which monitors and assesses the impact and safety profile of ART medicines in a cohort of patients [23].

One of the Centre’s main objectives is to determine and reduce morbidity and mortality related to ART drug use in HIV-infected patients [24]. From 2004, more than 1000 patients were enrolled into the Medunsa National Pharmacovigilance cohort in Tshepang clinic. In a report submitted to the NDoH in 2011, the MNPC noted that a large number of patients were LTFU at its sentinel sites, but the incidence and risk factors of LTFU were not known.

### Study population and sampling

HIV-infected patients who were more than 14 years of age and were on ART were selected to participate in this study using a systematic random sampling method at the PV sentinel site. Patients that signed informed consent were enrolled into the PV study. A retrospective case report form (CRF) was completed and submitted for data capturing after which patients were followed up prospectively with each clinic visit.

### Study design

This was a retrospective observational cohort study. Secondary data analysis of patients enrolled into the Medunsa PV cohort and being followed up at Tshepang clinic was performed. Chart reviews were done to include other variables that were not included on the standard PV CRF as well as to gather any missing information in the PV database.

### Definitions

The working definition of LTFU in South Africa is “when 3 calendar months have passed without the patient having drugs in hand” [25]. LTFU, as defined by Chi *et al. (2011),* is when a patient has had 180 days or more since the last clinic visit. This definition was found to minimise misclassifications of patients and was recommended for use to allow for comparison of programs [26]. In our study, a patient was classified as LTFU if the patient had been followed up at least once after ART initiation, but had not had contact with the clinic for 180 days or more since their last recorded expected return date, or if there were 180 days or more between the expected date of return and the next clinic visit. Patients that were not LTFU were referred to as patients that are “in care” in this article. A maximum of 3 months (90 days) of treatment can be dispensed to a patient at one time in Tshepang clinic. Therefore, according to our definition, patients that were classified as LTFU had not had treatment for 3 (if three months of treatment had been dispensed at the last clinic visit) to under 6 months (if less than 1 month of treatment had been dispensed to the patient at the last visit).

Virological suppression refers to an individual who has a fully suppressed viral load (VL), as evidenced by a VL level that is undetectable or a VL that is below 50 copies/ml.

A person who has a partner includes people who are married or are cohabiting with a life partner. A person without a partner includes people who are single, separated, divorced or who are a widow or widower and not living with or married to a partner.

An adverse event is “any untoward medical occurrence that may appear during treatment with a pharmaceutical product, but which does not necessarily have a causal relationship with the treatment” [20].

WHO Stages three and four in adults and adolescents (ages of 15 years or more) are stages that indicate advanced HIV symptoms and Severe advanced HIV symptoms, respectively, in people who have been found to be HIV infected, as illustrated by the presence of any clinical conditions or symptoms that are classified as WHO stage three or WHO stage four conditions [27].

Patients were classified as dead using the South African Home affairs website which identifies death status at the date and time of the enquiry. Retrospective information cannot be retrieved on the website therefore date of death could not be ascertained.

Patients that are clinically stable on ART and virologically suppressed are “down referred” to primary care facilities that are nearest to them, usually within the same sub-district, for patient follow-up [25].

Transferred patients are those patients that for any reason request to be followed up at a different clinic, often outside of the sub-district in which they were being followed up. They are given a transfer letter for further follow-up at their requested facility irrespective of whether they are clinically or virologically, well or unwell [25].

### Statistical analyses

Statistical analysis was done in STATA Corp version 12 [28]. Patients that were LTFU, transferred out, who had died and those who are still in care at Tshepang clinic were identified and described. Further analysis was then focused on the LTFU outcome. Patients’ data was censored on the 30th June 2012 and patients who were transferred out were considered to be still in care at the time of censoring. Patients who had missing files, an unknown initiation date or unknown last visit date or whose outcome could not be determined were not included in the analysis.

Medians, means and proportions were used to describe demographic characteristics and baseline characteristics. We compared the differences in variables measured at baseline and after ART initiation using chi2 for proportions and non-parametric statistics for data that was continuous and not normally distributed. Univariate comparisons between proportions of patients that were classified as LTFU and those not classified as LTFU was done using Kaplan-Meier survival curves with a log-rank test for equality of survival curves. Sex, age and factors that had a p-value less than 0.20 in bivariate analysis were included in the multivariable cox regression that was performed to determine significant predictors of LTFU. The sex and age variables were included into the model as known priori. A p-value of 0.05 or less was considered to be statistically significant.

### Ethical and legal considerations

All the participants in the MNPC cohort signed informed consent to be included in the PV surveillance study. Participants that were less than 18 years of age were required to provide verbal assent and their legal representative or guardian gave written informed consent for participation in the study. Ethical approval for this study was received from the University of Pretoria’s Faculty of Health Sciences Research Ethics Committee.

## Results

### Description of the Tshepang pharmacovigilance cohort

**Fig. 1:**
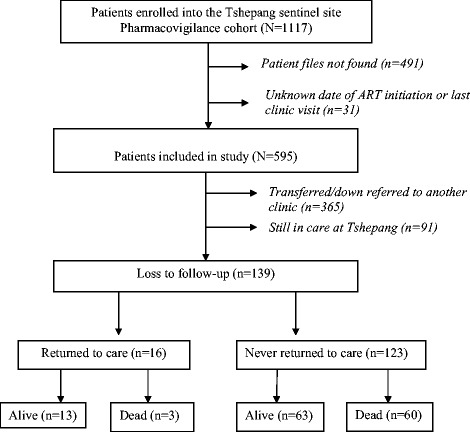
A flow diagram describing the patients enrolled into the Tshepang Pharmacovigilance cohort and their various outcomes, 2004–2012

Overall, 1117 patients were enrolled into the MNPC PV cohort at Tshepang sentinel site, from September 2004 to June 2012. Fifty-three percent (595/1117) of the patients were included in the current study. The other 522 (47 %) patients were excluded either because the patient file could not be retrieved from the clinic records (*n* = 491) or there was either an unknown date of ART initiation or unknown date of last clinic visit (*n* = 31) (Fig. 1). There were no significant differences in the distribution of sex (*p* = 0.334) and age (*p* = 0.891) variables in patients included in the study when compared to those excluded from the analysis.

Among the patients included in the analysis, 15.3 % (91/595) were still in care at Tshepang clinic, 61.3 % (365/595) had been down-referred or transferred to another clinic and 23.4 % (139/595) were LTFU. Of the patients who were LTFU, 11.5 % (16/139) returned to care after missing more than 180 days of clinic appointments and 10.6 % (63/139) were known to have died (Fig. 1). The proportion of patients that died was higher in patients that never returned to care (48.8 %) compared to those that returned to care (18.8 %), (*p* = 0.023). (Refer to Fig. 1). The median time in care for patients that were down referred or transferred was 614 days (Interquartile Range (IQR): 416 to 843 days). The median time in care for patients that are in care was 1205 days (IQR: 851 to 1604 days) and median time in care for those patients that were LTFU was 482 days (IQR: 251 to 1046 days).

### Baseline demographic characteristics of study participants

Table 1 shows the baseline demographic and clinical characteristics of the 595 patients that were included in the study. The median age at ART initiation was 35.9 years (IQR: 30.1- 43.5), and 65.5 % were women. At ART initiation the majority of the patients had achieved secondary level of education (60.6 %), were unemployed (75.5 %), and had no committed partner (80.3 %). Baseline demographic characteristics of patients that were LTFU versus those that remained in care were similar except with regards to employment status. A lower proportion of the patients that remained in care were unemployed (73.6 %) compared to the patients that were LTFU (81.0 %) (*p* = 0.028) (Table 1).

**Table 1:** Baseline demographic characteristics of patients included in study, Tshepang Pharmacovigilance cohort, 2004-2012

Variable		Total		LTFU		In care		χ^2^
		N	%	n	(%)	n	%	p-value
Sex	Female	390	(65.5)	87	(62.6)	303	(66.5)	0.402
Age at ART initiation (years)	≥ 45 years	129	(21.7)	30	(21.6)	99	(21.7)	0.113
	≥ 30 and < 45	320	(53.8)	66	(47.5)	254	(55.7)	
	< 30	146	(24.5)	43	(30.9)	103	(22.6)	
Racial grouping	Black	512	(99.4)	100	(99.0)	412	(99.5)	0.548
(80 missing)	Other	3	(0.6)	1	(1.0)	2	(0.5)	
Education level	None	34	(6.2)	7	(5.4)	27	(6.5)	0.110
(50 missing)	Primary	153	(28.1)	34	(26.2)	119	(28.7)	
	Secondary	330	(60.6)	77	(59.2)	253	(60.9)	
	Tertiary	28	(5.1)	12	(9.2)	16	(3.9)	
Employment status	Employed	141	(24.2)	24	(17.5)	117	(26.2)	0.028
(14 missing)	Unemployed	439	(75.3)	111	(81.0)	328	(73.6)	
	Self employed	3	(0.5)	2	(1.5)	1	(0.2)	
Live-in partner	Present	115	(19.7)	19	(14.0)	96	(21.4)	0.057
(10 missing)	Absent	472	(80.3)	117	(86.0)	353	(78.6)	

### Incidence of LTFU

**Fig. 2:**
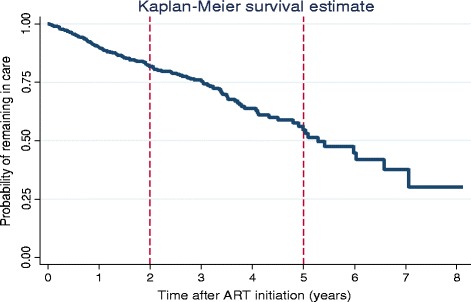
Kaplan Meier graph showing the probability of remaining in care with time, Tshepang Pharmacovigilance cohort, 2004–2012

The data of the 595 patients that we included in the study represented 1280 person-years of follow up time. The overall incidence rate of LTFU in the cohort was estimated to be 109 per 1000 person-years (95 % Confidence Interval (CI): 92–128). Among the patients LTFU, the median duration on follow up was 21.5 months (inter-quartile range (IQR): 12.9 - 34.7). Patient retention in care was 81.8 % at 2 years and 54.7 % at 5 years in the cohort as illustrated in Fig. 2.

**Fig. 3:**
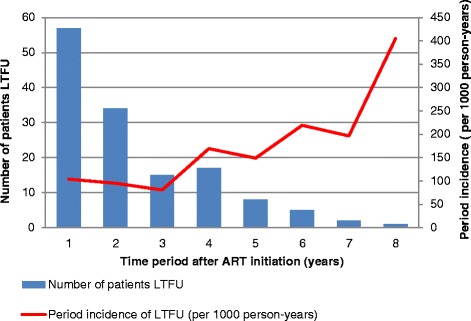
Number of patients and the period incidence of LTFU with each year of taking ART, Tshepang Pharmacovigilance cohort, 2004–2012

Approximately 40 % (57/139) of the patients that became lost to follow up were lost within the first year of being initiated on ART. The absolute number of patients that were LTFU with each year on ART appeared to decrease over time. However, on further analysis, we found that the incidence rate of LTFU was 103 per 1000 person-years in the first year on ART and increased to 405 per 1000 person-years in the eighth year of taking ART (Fig. 3).

### Clinical characteristics of the participants

The baseline patient weight was lower (median = 55.6 kg) among patients who became LTFU, compared the baseline weight (median = 57.2 kg) of patients who remained in care (p < 0.001). The median patient weight increased from 55.6 kg at baseline to 58.5 kg at the last recorded weight among patients LTFU (p < 0.001), and from 57.2 kg at baseline to 65.2 kg at the last clinic visit among patients who remained in care (p < 0.001). Median of the last known weight was lower in patients that were LTFU (58.5 kg) than those that were not (65.2 kg) (*p* < 0.001) (Table 2).

**Table 2:** Characteristics of patients that were LTFU compared to those that were not LTFU, Tshepang Pharmacovigilance cohort, 2004-2012

Variable	LTFU		In care		Rank sum p-value
	Median	IQR	Median	IQR	
Baseline weight (kg)	55.6	46.8 - 62.6	57.2	51.0 - 66.0	<0.001
Last known weight (kg)	58.5	48.5 - 66.7	65.2	56.8 - 73.3	<0.001
Baseline CD4 count (cells/ml)	79.0	30.0 - 146.0	87.0	36.0 - 150.0	<0.001
Last known CD4 count (cells/ml)	197.5	113.8 - 347.0	275.0	177.5 - 389.2	<0.001

Baseline CD4 cell counts were lower among patients who later became LTFU (median = 79 cells/ml, IQR: 30–146), compared to cell counts for patients who remained in care (median = 87cells/ml, IQR: 36–150) (*p* < 0.001) (Table 2). This was the same for last known CD4 count where median CD4 count was significantly lower in patients that were LTFU (median = 197.5, IQR: 113.8-347.0 cells/ml) than in those that were not LTFU (median = 275.0, IQR: 177.5-389.2cells/ml). The median CD4 counts increased from 79 cells/ml at baseline to 198 cells/ml at the last measured count among patients LTFU (*p* < 0.001), and increased from 87 cells/ml at baseline to 275 cells/ml at last measured count among patients who remained in care (*p* < 0.001) (Table 2).

**Fig. 4:**
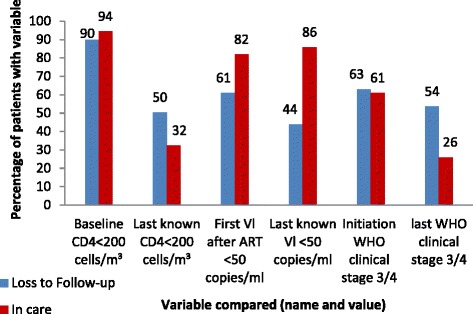
Comparing CD4 counts, viral load levels and clinical staging in patients that were LTFU and that are in care, Tshepang Pharmacovigilance cohort, 2004–2012

There was a lower proportion (121/135, 90 %) of patients with CD4 count <200 cells/ml at baseline measurement in patients that became LTFU than in those that were not LTFU (427/452, 94.4 %) (*p* = 0.048). There was, however, a higher proportion (60/118, 50.8 %) of patients that had a last known CD4 count <200 (*p* < 0.001) in patients that were LTFU (60/119, 50.4 %) when compared patients that were not LTFU (43/441, 32.4 %) (Fig. 4).

Most (62 %) patients were in WHO clinical stages three or four prior to ART initiation. At the initiation of ART, 63 % (80/127) of the patients that became LTFU were in WHO clinical stages 3 or 4, compared to 61 % (255/418) of the patients that remained in care (*p* = 0.190). However, at last clinic visit before outcome, 53.6 % (67/125) of patients that were LTFU had a WHO clinical stage 3 or 4, compared to 26.0 % (100/384) of the patients that remained in care (*p* < 0.001) (Fig. 4).

Sixty-one percent (61/100) of the patients that were LTFU had an undetectable VL when it was first measured after initiating ART compared to 81.9 % (353/431) of patients that remained in care (p < 0.001). At last recorded VL measurement, 43.8 % (50/114) of the patients that were LTFU had an undetectable VL compared to 85.8 % (376/438) of patients that remained in care (*p* < 0.001). The findings show a decrease in the percentage of patients that had undetectable VL levels among patients that were LTFU (*p* = 0.012), while revealing no significant changes in the percentage of patients with undetectable VL among patients who remained in care (*p* = 0.114).

### Treatment and related characteristics

Most patients in the cohort (581/591, 98.3 %) were initiated on stavudine (d4t) containing regimens. Almost 18 % (100/568) of patients reported that they drank alcohol at the time of ART initiation. A total of 316/567 (55.7 %) patients in the cohort have experienced at least one ART related adverse event. There were no differences in the proportions of patients who were initiated on a stavudine containing regimen (*p* = 0.466), drank alcohol at baseline (*p* = 0.852) or experienced an ART related adverse event during treatment (p = 0.057), when comparing patients that were LTFU to those that remained in care (Table 3).

**Table 3:** ART and adverse event related characteristics of patients that were LTFU compared to those that remained in care, Tshepang Pharmacovigilance cohort, 2004-2012

Variable		Total		LTFU		In care		χ^2^
		N	%	n	(%)	n	%	p - value
Initiating regimen	d4t containing*	581	98.3	443	98.0	138	99.3	0.466
4missing	Other	10	1.7	9	2.00	139	0.7	
Alcohol	No	468	82.4	107	82.95	361	82.2	0.852
27 missing	Yes	100	17.6	22	17.05	78	7.8	
Adverse events	No	251	44.3	67	51.5	184	42.1	0.057
28 missing	Yes	316	55.7	63	48.5	253	57.9	

*d4t – stavudine

Close to a quarter (74/316, 23.4 %) of patients that had adverse events experienced more than one adverse event. The most commonly reported adverse events included peripheral neuropathy (165/316, 52.2 %), lipodystrophy (121/316, 38.3 %) and hyperlactaemia (83/316, 26.3 %). Other ART related adverse events that were experienced included anaemia, various dermatological adverse events, central nervous system adverse events, diarrhoea and vomiting, lipid abnormalities and hepatitis. The proportion of LTFU patients who experienced peripheral neuropathy was 28.1 % (39/139), along with 7.2 % (11/139) who experienced lipodystrophy, 9.4 % (13/139) experienced hyperlactataemia, and 31.6 % (6/139) experienced other adverse events (Table 4).

**Table 4:** ART related adverse events experienced by patients and proportions that had adverse events in patients that were LTFU versus those still in care, Tshepang Pharmacovigilance cohort, 2004-2012

Adverse event	Total		LTFU		In Care	
	N	%	n	(%)	n	%
Peripheral Neuropathy	165	52.2	39	28.1	126	21.2
Lipodystrophy	121	38.3	11	7.2	110	24.1
Hyperlactateaemia	83	26.3	13	9.4	70	15.4
*Others	19	6.0	6	4.3	13	2.9

*Others include anaemia, various dermatological adverse events, Central nervous system adverse events, diarrhoea and vomiting, lipid abnormalities and hepatititis

### Factors associated with LTFU

In the multivariable Cox regression model, the risk factors that were independently associated (*p* < 0.05) with becoming LTFU were not having a committed partner (aHR = 2.88, 95 % CI 1.19-6.97), being self-employed (aHR = 13.92, 95 % CI: 2.81-69.06), having a baseline CD4 count > 200 cells/ml (aHR = 3.81, 95 % CI: 1.85-7.85), having a history of an ART related adverse event prior to outcome (aHR = 0.61, 95 % CI: 0.38-0.99), having a last known viral load that was detectable (aHR = 3.60, 95 % CI: 1.98-6.52), and having a last known WHO clinical stage three or four (aHR = 2.00, 95 % CI: 1.22-3.27), (Table 5). Education level and alcohol intake status at ART initiation, regimens on which patients were initiated and baseline WHO clinical staging were not associated with becoming LTFU on univariate analysis and were not included in the multivariable regression model (Table 5).

**Table 5:** Cox Regression analysis to determine factors associated with LTFU, Tshepang Pharmacovigilance cohort, 2004-2012

Characteristic		Univariate analysis			Multivariate Analysis		
		HR	95 % CI	P value	aHR	95 % CI	P value
Age at ART	≥ 45	Ref*			Ref*		
Initiation	≥ 30 and < 45	0.77	0.50 - 1.19	0.240	0.71	0.37 - 1.37	0.310
	< 30	0.94	0.59 - 1.51	0.810	0.58	0.28 - 1.21	0.144
Partner status	Present	Ref*			Ref*		
	Absent	1.75	1.08 - 2.84	0.024	2.88	1.19 - 6.97	0.019
Employment	Employed	Ref*			Ref*		
Status	Self employed	7.18	1.69 - 30.54	0.008	13.92	2.81 - 69.06	0.001
	Unemployed	1.57	1.01 - 2.44	0.045	1.75	0.86 - 3.56	0.123
Adverse events	No	Ref*			Ref*		
	Yes	0.49	0.34 - 0.70	<0.001	0.61	0.38 - 0.99	0.044
Baseline CD4	CD4 ≤ 200	Ref*			Ref*		
Count	CD4 > 200	2.47	1.42 - 4.31	0.001	3.81	1.85 - 7.85	< 0.001
First VL level	Undetectable VL	Ref*			Ref*		
after ART	Detectable VL	1.81	1.21 - 2.72	0.004	1.00	0.58 - 1.73	0.998
Last known CD4	CD4 ≤ 200	Ref*			Ref*		
Count	CD4 > 200	0.42	0.29 - 0.60	< 0.001	0.88	0.51 - 1.53	0.651
Last known VL	Undetectable VL	Ref*			Ref*		
Level	Detectable VL	3.20	2.20 - 4.67	< 0.001	3.60	1.98 - 6.52	< 0.001
Last known WHO	Stage 1 or 2	Ref*			Ref*		
clinical stage	Stage 3 or4	3.20	2.24 - 4.57	< 0.001	2.00	1.22 - 3.27	0.006

*Ref - Reference category

## Discussion

We found a progressive increase in the incidence of LTFU patients with each year after initiation of ART: the patient retention rate was 82 % after 2 years and 55 % after 5 years. This finding is similar to other studies done in sub-Saharan Africa [8–10]. It is, however, important to keep in mind that different definitions of LTFU may have been used in these studies. Due to limited research, we compare our study findings to the available research literature, bearing in mind that some of these study findings may or may not have been different, if our definition of LTFU would have been applied to their analysis. Our retention rate at 2 years gives the impression that our retention rates are at higher end of the rates that have been reported in some sub-Saharan ART programs (range from 24 to 77 % at 2 years) [8].

South Africa has a goal that at least 70 % of patients that are initiated on ART are alive and on treatment 5 years after ART initiation [11]. Though we appear to have comparatively better programmatic success in retaining patients in care at 2 years after ART initiation when compared to other sub-Saharan programs, we are not achieving this 70 % retention target at 5 years after ART initiation, and more effort is needed to improve patient retention in care at 5 years after ART initiation.

Our study, like other studies that have shown that being single is a risk factor for becoming LTFU [20], found that patients that did not have a committed partner had a higher risk of becoming LTFU. This suggests that having a committed primary relationship is protective against being LTFU. In our study we found that 80 % of patients did not have committed primary relationships. It is therefore important that other systems and programs that lower the rates of LTFU be employed. Integrated patient care, community based adherence support programs, active outreach to patients, the availability of adherence support services as well as facility and community based patient adherence groups on ART have been found to lead to lower rates of LTFU [12, 14–16, 19, 20]. These findings are well in support of the South African government’s call for integrated patient care and the nationwide rolling out of adherence clubs/groups. It is also important for health facilities to investigate facility specific factors that can impact LTFU rates and implement programs that are tailored specifically to reduce LTFU in their unique settings [19]. Though other studies have also shown that young age, being male and illiteracy are risk factors for becoming LTFU, an association between age, sex or level of education and becoming LTFU was not found our study [9, 17, 20].

The benefits of taking ART were clearly identified: we found that there were significant increases in weight as well as in immune function (evidenced by increasing CD4 cell counts) from the time of ART initiation till the last known measurements both in patients that were LTFU and in those that remained in care. Our findings showed that patients that became LTFU had significantly lower weight and CD4 cell count measurements both at ART initiation and at last known measurement. However, patient weight was not associated with becoming LTFU.

There have been varying findings with regards to the association between baseline CD4 cell count and LTFU, with some studies showing that patients that had CD4 counts between 301 cells/ml and 350 cells/ml had a lower risk of becoming LTFU than those with CD4 counts < 200 cells/ml [13], while others showed that higher baseline CD4 cell count was associated with a higher risk of LTFU [12, 20, 21]. Our findings showed that patients that had a CD4 cell count of > 200 cells/ml at ART initiation were at a higher risk of becoming LTFU. This finding could possibly be explained by the fact that patients with a high CD4 cell count generally do not have the typical clinical symptoms of advanced HIV disease and thus, may not perceive the benefits of the strict patient follow-up schedule. However, we did not find any association between baseline WHO clinical staging and LTFU to further support this explanation. That we did not find any significant association between baseline WHO staging and LTFU was unlike other studies [17]. This could be explained by the fact that eligibility criteria for ART initiation may be based only on WHO staging in resource constrained countries, and may account for the variable study findings regarding the association between LTFU and WHO clinical staging. The use of WHO clinical staging as an ART eligibility criteria, may not necessarily correlate with immune status (CD4 count) or actual disease progress, especially where diagnostic tests for AIDS defining conditions are not readily available.

In our study we were able to demonstrate that patients that were not virologically suppressed (VL > 50 copies/ml) at last clinic visit had a three times higher risk of becoming LTFU than those that had a suppressed VL. Similarly, patients who had advanced clinical disease at last clinic visit (WHO clinical stage three or four) were twice as likely to become LTFU. Similar findings have been reported in other studies [17, 18]. It is important for measures to be put in place that improve retention rates among patients whose viral loads remain high and/or who continue to display advanced clinical stages of HIV disease, despite taking ART.

There is limited literature on the association between ART adverse events and LTFU. Just over half (56 %) of our cohort had a history of at least one ART related adverse event since ART initiation. Less than a quarter of the patients who reported experiencing each of the common ART related adverse event became LTFU. We would have expected that patients experiencing adverse events would likely be discouraged from taking their ART. However we found that these patients had a lower risk of becoming LTFU when compared to patients that had not had ART related adverse events. This result may possibly be due to the perception that patients have of their health status, but further investigation is necessary.

The MNPC is the only structured surveillance cohort that has been set up by the South African National Department of Health to follow up patients that have been initiated on ART. As such, the cohort is the evidence base which provides insight into different aspects of the National ART program. Our study did have limitations that should be taken into account when interpreting the findings. Firstly, we only analysed about 53 % of the patients that were enrolled into the PV surveillance cohort during the period of the study. This could have possibly introduced some selection bias into the study and could have led to an over- or under-estimation of the true incidence of LTFU in our study. We, however, found no significant differences in demographic characteristics when we compared the patients that we included in the study and those that were excluded. Secondly, we were unable to obtain the date of death for some of the patients that died after being LTFU, and thus were not able to confirm whether the patients died 180 days or more after the last clinic appointment. It is therefore possible that some of these patients were misclassified as LTFU, resulting in an over-estimation of the true incidence of LTFU. Thirdly, we assumed that all the patients that were transferred from Tshepang clinic to other ART clinics were still in care at the time of censoring. It is possible that some of these patients became LTFU after being transferred, leading to an under-estimation of the rate of LTFU in our study. Lastly, it is also likely that some of the patients that we classified as LTFU could have self-transferred and continued ART at other sites without notifying Tshepang clinic. This could have led to an over-estimation of the incidence rate of LTFU in our study. Despite these limitations, we believe that our study findings provide an important insight into the magnitude and the factors related to LTFU among patients on ART.

## Conclusions

Our study shows that LFTU after ART initiation is high in South Africa, although patient retention in care at 2 years after ART initiation appears to be higher than in some ART programs in sub-Saharan Africa. The incidence rate of LTFU increases with additional years on ART, and patient retention at 5 years is much lower than the target of at least 70 % that is in the South African NSP on HIV, STI’s and TB (2012–2016). Our findings will help ART clinicians to recognize patients that require additional support to remain in care, such as patients with higher CD4 cell counts (> 200 cells/ml) at baseline, patients who do not have committed partners and patients who continue to have detectable VL (> 50 copies/ml) or advance clinical disease (WHO clinical stages 3 or 4) after ART initiation. These patients require additional support such as community based adherence support programs and the use of reminder tools, which have been shown to reduce LTFU in similar settings. There is also a need to intensify surveillance activities in order to allow the country to accurately monitor rates of, and reasons for, LTFU in patients that are on ART.

## Abbreviations

ART: Antiretroviral Therapy
CI: Confidence Interval
CRF: Case Report Form
d4t: Stavudine
HIV: Human Immunodeficiency Virus
HR: Hazard Ratio
aHR: Adjusted Hazards Ratio
IQR: Interquartile Range
LTFU: Loss To Follow-Up
MNPC: Medunsa National Pharmacovigilance Centre
NDoH: National Department of Health
PV: Pharmacovigilance
UNAIDS: United Nations program on HIV/AIDS
VL: Viral Load
WHO: World Health Organisation
